# Recent Advances in Cereal Arabinoxylans: A Review of Extraction, Processing and Structure Relationships with Advanced Applications

**DOI:** 10.3390/foods15111905

**Published:** 2026-05-28

**Authors:** Wenda Liu, Shiyu Xu, Zijie Lu, Xiaoqi Xu, Sha Li, Hong Xu

**Affiliations:** College of Food Science and Light Industry, Nanjing Tech University, Nanjing 211800, China

**Keywords:** green extraction, oxidative gelation, ferulic acid crosslinking, cereal bran valorization

## Abstract

Arabinoxylans (AX) are the main hemicellulose polysaccharides found in cereal bran and endosperm. These polysaccharides have attracted widespread attention owing to their potential as both functional components and structural building materials. Previous reviews typically discussed AX extraction, arabinoxylan oligosaccharides preparation, dietary fiber function, or gelation behavior separately. This review aims to address this limitation by systematically reviewing relevant research from an integrated “structure–processing–function” framework. Specifically, this review compares the effects of different extraction pathways on yield and key structural features, including molecular weight, substitution patterns, and ferulic acid retention. Moreover, the review summarizes the roles of physical, chemical, and enzymatic modifications in regulating solubility, interfacial behavior, and gelling ability. The review further discusses the effects of these structural changes on the application of AXs in food, delivery systems, and some biomaterials. Mild or enhanced assisted extraction is conducive to maintaining structural integrity, whereas high-yield processes are often costly. This often manifest as a decrease in depolymerization and ferulic acid acylation. Correspondingly, oxidative gelation and complex network design have expanded the functional applications of AXs. However, its final performance remains substantially limited by differences in raw material sources, processing conditions, and the balance between covalent and noncovalent interactions. Therefore, AXs should be considered promising but not yet controllable functional polysaccharides. Major bottlenecks in this field include structural heterogeneity from different grain sources, insufficient control of modification results, incomplete understanding of the structure–function relationship, and a lack of sufficient clinical and regulatory support for some potential applications. Only by making substantive progress on these key issues can AXs transform into a stable system of high-value-added food and biological materials.

## 1. Introduction

Agricultural processing generates a large number of by-products and waste logistics, which exerts dual pressure on the environment and the economy. In the context of climate change, sustainable food production and circular bioeconomy, the effective utilization of agricultural food by-products has become an important strategy for recycling functional components and developing high value-added food materials. Food by-products are usually rich in dietary fiber, protein, lipids, phenols and other bioactive components, which can be further developed into food ingredients, packaging materials, and related products [1,2]. Grain bran has a large output in grain processing and is rich in polysaccharides with important structural and functional value. However, grain bran remains mainly used in low value fields, such as animal feed and fertilizer [3]. Therefore, improving the utilization level of grain bran is essential for sustainable resource utilization and direct food industry innovation.

Arabinoxylan (AX) is one of the most important non-starch polysaccharides in wheat, rye, and barley brans, and its structure consists of a β-D-xylose (Xyl) backbone and an α-L-arabinose (Ara) side chain [4]. Some arabinose residues can be esterified with ferulic acid, which is closely related to antioxidant activity, postprandial blood glucose regulation, oxidative gel formation, and gut microbiota regulation [5]. These properties render AXs multifunctional food ingredients with both nutritional and structural functions. AXs have been approved or accepted for use in specific food applications. In particular, in the United States, a Generally Recognized as Safe notice was issued for the application of corn bran AX for specific conventional food applications. Moreover, in China, AX derived from sugarcane bagasse has been approved as a new food ingredient.

**Figure 1:**
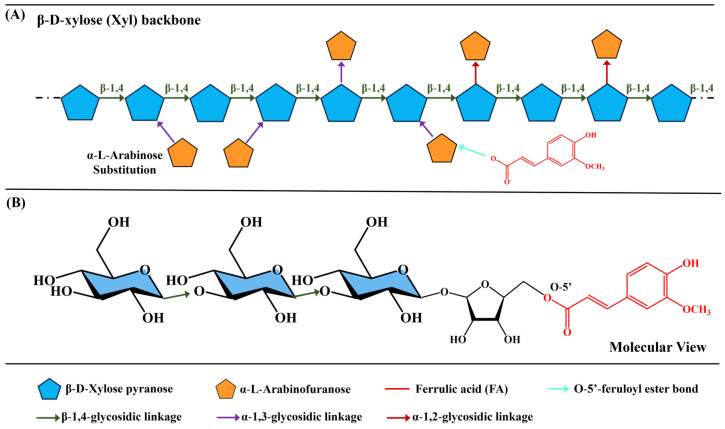
Schematic representation of the arabinoxylan structure, showing the β-(1→4)-xylan backbone, arabinose substitutions, ferulic acid esterification, and the main enzymatic cleavage sites.

Structurally, AXs have a linear backbone composed of β-(1→4)-D-xylpyranose residues connected to β-L-arabinofuranose side chains. The arabinose residues can be monosubstituted with O-2 or O-3, disubstituted with O-2 and O-3 on the same xylose unit, or remain unsubstituted (Figure 1A). The A/X (Ara/Xyl) ratio and substitution mode collectively determine the degree of branching and conformational flexibility of the chain [6,7,8]. Another key feature is the esterification modification of ferulic acid at the O-5 site of specific arabinofuranose residues (Figure 1B). Under enzymatic or chemical oxidation conditions, these ferulic acyl groups undergo oxidative coupling, thereby connecting the AX chains into a three-dimensional network and subsequently forming the structural basis for the formation of AX gel [9]. Therefore, the function of AXs mainly depends on the substitution mode, molecular weight, and ferulic acid content. In general, components with a high degree of branching tend to have enhanced water solubility and solution viscosity, whereas components with large chain length and a high degree of ferulic acylation are conducive to the formation of oxidative networks.

Previous studies have discussed AX extraction, arabinoxylan oligosaccharides (AXOS), dietary fiber function, and gelation behavior [10,11,12]. However, the relationship between structural regulation, gelation behavior, and application performance lacks sufficient integration. Therefore, this review examined cereal AXs from a framework of structure processing and function. Moreover, this review compared the main extraction pathways and their impact on yield and key structural parameters. The review also summarizes physical, chemical, and enzymatic modification strategies and further discusses the effects of these changes on gel formation and their end performance in food and materials. Through this organization, this review aimed to go beyond broad descriptive summaries and critically analyze the main trade-offs, opportunities, and limitations that determine the effective utilization of cereal AXs.

## 2. Comparative Extraction Techniques of AXs

### 2.1. Alkali Extraction

Alkali extraction is one of the most widely used methods for AX extraction owing to its ability to break the ester bond and hydrogen bond interactions in the cell wall matrix. However, its extraction effect depends on the treatment intensity and the type of raw material. As shown in Table 1, AX obtained from alkali-treated corn bran had a relatively low A/X ratio (0.51) but a high molecular weight (770 kDa) [13]. This indicated that its degradation was limited. In contrast, strong treatment increased the yield to 31.1% in wheat bran but reduced the molecular weight to 263 kDa [14]. Mild extraction conditions attained a low yield (11.0%) but preserved the molecular weight of the polymer (310 kDa) [15], whereas the NaOH/urea system showed a balanced result by achieving a yield of 16.4% while maintaining a molecular weight of 297 kDa and ferulic acid content of 44.88 mg/g [16]. These results indicated that high yields do not necessarily translate to superior functional quality. The AX fraction that retained large molecular sizes and ferulic acid increased viscosity, promoted oxidative cross-linking, and formed gels. In contrast, the severely degraded components were primarily suitable as soluble dietary fiber ingredients [17]. From an industrial perspective, alkaline extraction also has critical limitations, including high chemical and water requirements, alkaline wastewater generation, equipment corrosion, and poor compatibility with food production processes [18]. However, strong alkaline conditions and improved recovery rates may result in the loss of structural characteristics required for high value applications.

### 2.2. Water Extraction and Subcritical Water Extraction

Water extraction is the gentlest way to separate AXs. The process mainly recovers the water-extractable arabinoxylan (WEAX) component originally present in cereal bran. The main advantage of water extraction is reduced damage to the natural polymer structure, thereby rendering it conducive to obtaining relatively intact AXs with a high molecular weight. Water extraction mainly extracts soluble components; however, the yield is usually low. As shown in Table 1, the AX yield of wheat bran after water extraction was only 9.3%; however, the obtained component maintained a high molecular weight (403 kDa) [19]. Moreover, the molecular weight of AXs obtained from oat bran was even higher (513 kDa) than that obtained from wheat bran [20]. Therefore, water extraction is most suitable for obtaining WEAX with good structural preservation, rather than obtaining the maximum recovery rate of total AXs. To improve the accessibility of the matrix, pretreatment steps, such as destarching or physical crushing, are usually combined.

Subcritical water extraction (SWE) provides an efficient hydrothermal release pathway. Under high temperature and high-pressure conditions, SWE improves solvent diffusion, weakens intermolecular interactions in the cell wall, and promotes the release of AXs, including some AXs bound to the cell wall. Compared with that under conventional water extraction, the yield of SWE was markedly improved, reaching 27.5%, 29.4%, and 40.5% in wheat bran, barley bran and rye bran, respectively [21]. However, the yield increase was accompanied by a pronounced decrease in molecular weight, with the obtained AXs dropping to 90–200 kDa, thus indicating partial depolymerization [22]. The source of raw materials also affected the extraction results. In particular, rye bran had the highest yield, but the obtained AXs had a low molecular weight, whereas wheat bran had a low yield but retained a relatively large polymer component. Under the conditions of pH 7 and 160 °C, SWE improved the purity of AXs to a certain extent and retained some ferulic acid substitution. Overall, water extraction was best suited for recovering relatively intact WEAX, whereas SWE was best suited for achieving a broad range of AX recovery. However, SWE usually reduced the integrity of the molecular structure.

### 2.3. Enzyme-Assisted Extraction

Enzymatic extraction employs specific biocatalysts to selectively break down the cell wall polysaccharide structure under mild conditions. This releases AXs and highly soluble AXOSs, thereby preserving ferulic acid integrity and antioxidant capacity. Zhang et al. [23] revealed that targeted enzymatic treatment, especially xylanase-based processing, generated isolates with higher purity and fewer by-product impurities than those under chemical methods [23].

Mechanistically, different enzymes cleave xylan in different locations. Endo-1,4-β-xylanase specifically hydrolyzes β-1,4-D-xylosidic linkages within the xylan backbone, whereas α-L-arabinofuranosidase targets arabinose side chains, usually at the O-2 or O-3 positions of xylose residues. The specificity of the enzyme allowed the main chain to have multiple depolymerization mechanisms to regulate AX structure [24]. The data in Table 1 shows that AX fractions differ in yields and molecular traits depending on substrate and enzyme. Enzyme-assisted treatment of wheat bran yielded only 7.6% AX (A/X = 0.54), thereby suggesting that most AXs remained bound in the bran. This indicated that AXs were tightly embedded in the bran matrix and only a limited component may have been released. Nevertheless, food-grade enzymes are relatively expensive, and large-scale processing requires strict control to maintain the reproducibility of enzyme activity [25]. Moreover, downstream enzyme inactivation or removal may add additional steps [26]. Overall, enzymatic extraction is a mild method that meets food safety standards. However, its industrial value depends on whether the improved purity and selectivity are sufficient to offset the increased costs, processing time, and complexity of large-scale production.

### 2.4. Ultrasonic Extraction

Ultrasound-assisted extraction (UAE) effectively disrupts plant cell wall structural integrity by using micro-jet and strong shear force generated by acoustic cavitation. The process promotes solvent penetration and enhances the dissolution of polysaccharides, thereby improving the recovery rate of AXs. UAE also shortens the extraction time and greatly reduces solvent consumption. The instantaneous high pressure generated by the collapse of cavitation bubbles rapidly decomposes the cell–matrix and promotes efficient release of attached macromolecules.

The AX yields are substantially improved by optimized UAE conditions. Jiang et al. revealed that applying 0.30 M NaOH for 25 min at 500 W yielded water-insoluble arabinoxylan (WUAX) 27.88% (A/X 0.74). Notably, the response to ultrasonic power was nonlinear. When the power increased from 200 W to 500 W, the yield decreased from 21.64% to 25.39%. A further increase to 600 W markedly reduced recovery [27]. Other studies also obtained similar recovery levels at moderate ultrasonic power, and the AX yield was close to 28% [28]. A similar trend was also observed in the extraction of beer lees AXs [29]. In addition, large-scale application remained limited by uneven energy distribution and the difficulty of process control. These results suggested that moderate ultrasound effectively achieved cell wall destruction and polysaccharide release. However, excessive power led to rapid degradation, thereby reducing extraction effectiveness.

### 2.5. Microwave Extraction

Microwave extraction uses dielectric heating to rapidly raise the temperature of the aqueous solution system to accelerate cell wall destruction and AX release. This effectively overcomes the thermal gradient limitation associated with traditional conduction heating [30,31]. Although the final effect depends on treatment intensity, the method substantially shortens treatment time and improves extraction efficiency.

Amutha Gnana Arasi et al. showed that fine-tuning microwave power and irradiation time accelerated matrix destruction. Moreover, these conditions created favorable conditions for subsequent quality transfer [32]. In general, increasing temperature or prolonging the irradiation time usually promotes AX release by promoting matrix decomposition and solvent permeation. However, harsh conditions may severely damage the integrity of macromolecules. Roos et al. reported that 11% AXs could be obtained via microwave-assisted treatment of barley hulls at 180 °C for 15 min. The A/X ratio was 0.60 and the molecular weight was 31 kDa [33]. A similar phenomenon of AX and AXOS components transferred to small molecules was observed in brewer’s grains under microwave-superheated or dilute alkaline conditions [34]. This indicated that expanding the extraction yield under enhanced conditions was bound to be accompanied by substantial depolymerization. Therefore, although microwave treatment offered advantages, such as rapid heating and high energy efficiency, the industrial scale still required strict control of heating to avoid localized overheating and inconsistent product quality.

### 2.6. Comparison and Prospects of Extraction Methods

**Figure 2:**
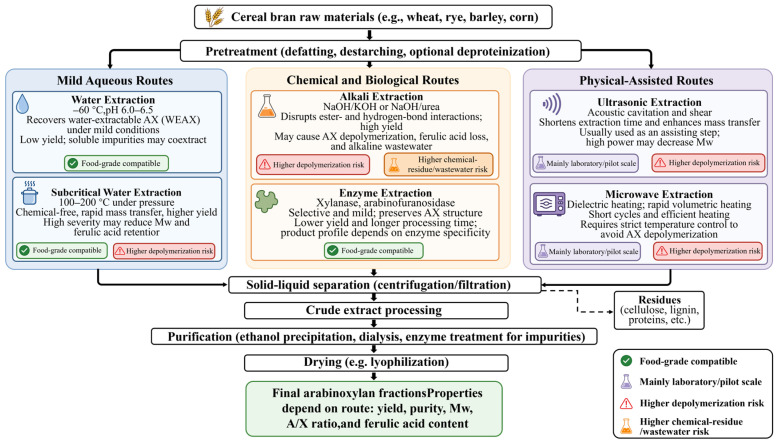
Main extraction routes for cereal arabinoxylans (AXs), showing their representative features, processing steps, and major trade-offs in structural preservation, food-grade compatibility, and processing burden.

Figure 2 shows that the main AX extraction routes, including chemical, physical-assisted, enzymatic, and water-based extraction, differ in yield, structural preservation, environmental impact, and economic feasibility. Although alkali extraction had high efficiency in the recovery of WUAX, it brought considerable burden to the environment. Physically assisted technology shortened the extraction time and substantially retained bioactive components; however, it re-quired extensive equipment investment and strict process control. Enzymatic extraction and water extraction were performed under mild food-grade conditions with excellent safety. However, they were usually limited by low recovery rates and high operating costs.

**Table 1:** Extraction conditions, yield, and key structural parameters of AXs obtained from different cereal brans. Note:/indicates that it was not mentioned in the text.

Extraction Method	Raw Material	Key Extraction Conditions	Yield/%	A/X	*M*_w_/kDa	Ferulic Acid Content/(mg·g^−1^)	References
Alkali extraction	Corn bran	0.75 M NaOH, solid–liquid ratio 1:20 (*w*/*v*), at 50 °C, 3000 rpm, for 20 min	/	0.51	770	/	[13]
Alkali extraction	Wheat bran	0.5 M NaOH, solid–liquid ratio 1:8 (*w*/*v*), at 80 °C, for 16 h	31.1	0.70	263	/	[14]
Alkali extraction	Wheat bran	1 M NaOH, solid–liquid ratio 1:20 (*w*/*v*), at 25 °C, for 15 h	11	0.71	310	/	[15]
Alkali extraction	Wheat bran	1.56 M NaOH, 1.36 M CO(NH_2_)_2_, solid–liquid ratio 1:20.23 (*w*/*v*), at 25 °C for 3 h	16.4	/	297	44.88	[16]
Water extraction	Wheat bran	pH to 6.0 with hydrochloric acid, solid–liquid ratio 13:90 (*w*/*v*), at 60 °C, for 30 min	9.3	0.48	403	3	[19]
Water extraction	Wheat bran	2.5 M CH_3_OH, sonicated for 1 h	3.1–4.5	/		3.51–4.91	[35]
Water extraction	Oat bran	6.52 M CH_3_CH_2_OH stand overnight at 4 °C for 12–16 h	/	0.40	513	/	[20]
Subcritical water extraction	Wheat bran	50 mM Na_3_PO_4_ buffer, pH to 7.0, solid–liquid ratio 1:10 (*w*/*v*), at 160 °C, for 15 min	31	0.35	130	13.50	[22]
Subcritical water extraction	Rye Bran	At 160 °C, pH to 7, for 15 min	40.5	0.27–0.48	100	9.50	[21]
	Barley bran		29.4	0.47–0.68	90	8.20	
	Wheat bran		27.5	0.32–0.46	200	7.30	
Enzyme extraction	Wheat bran	*Bacillus amyloliquefaciens* (BaA) and *Bacillus licheniformis* (BlA), at 90 °C, 140 rpm, for 1.5 h	7.6	0.54	/	/	[36]
Enzyme extraction	Corn bran	5 μL endoxylanase solid–liquid ratio 1:20 (*w*/*v*), at 55 °C for 24 h	/	/	93	/	[37]
Ultrasonic extraction	Wheat bran	0.30 M NaOH ultrasonic power: 500 W, solid–liquid ratio 1:30 (*w*/*v*), at 70 °C, for 25 min	27.8	0.74	/	/	[27]
Microwave extraction	Barley bran	60 mL H_2_O, solid–liquid ratio 1:6 (*w*/*v*), at 180 °C for 15 min	11	0.54	/	/	[33]
Microwave extraction	Corn bran	Microwave-assisted autohydrolysis, 160–200 °C for 2–20 min; optimum at 180 °C for 10 min or 200 °C for 2 min	46.3	0.47	/	6.62	[30]

In the future, the progress of AX extraction technology may depend on the synergistic strategy of integrating complementary technologies. For example, SWE can be combined with extrusion pretreatment. Moreover, retaining the ferulic acid group and high molecular weight structure maximized yield. In addition, the use of green solvents, particularly deep eutectic solvents, replaces conventional alkaline systems to reduce wastewater and environmental impact. An-other strategy is to develop precise extraction methods to obtain pure AX fractions with high purity and structural uniformity, such as highly branched fractions or fractions with specific molecular-weight distributions. These precise extraction methods substantially enhance the functional specificity and application potential of individual AX fractions.

## 3. Structural Modification of AXs

### 3.1. Structural Limitations and Functional Tailoring

In cereal brans, native AXs shows low solubility and complex structure, which prevents consistent gelation and controlled use. Dorjević et al. suggested that high molecular weight and complex branched chain configuration may improve the viscosity of the system but also reduce the conformational flexibility of the chain segment and masked some functional sites [38]. Therefore, the focus of current research was no longer on simply describing the natural structure of AXs, but rather on the directional regulation of AXs through physical, chemical, and enzymatic methods. The aim was to achieve synergistic optimization of solubility, gelation behavior, and target biological activity.

### 3.2. Physical Modification

Physical modification mainly alters the chain length and aggregation state of AXs without introducing additional chemical reagents; however, structural regulation is generally less selective than that of enzymatic or chemical methods. Extrusion treatment induces partial chain breakage and reduces intermolecular entanglement through heat and shear, thus converting high-molecular-weight AXs into small components, improving solubility, and reducing viscosity. For example, the average molecular weight of rye AXs decreased from 520 to 234 kg/mol after extrusion, whereas its solubility increased, its viscosity decreased, and it altered the ecotypes of various gut microbiota [39]. The main advantages of this method was ease of continuous processing and scale up. Moreover, the method had good compatibility with food processing; however, excessive shearing may also lead to over-degradation and weaken the gelation potential.

Electron beam irradiation mainly modifies AXs via dose-dependent chain scission. Chen et al. [40] reported that the number-average molecular weight of water-insoluble AX decreased to 199.49 kDa, and the weight-average molecular weight reduced to 234.36 kDa. Moreover, the solubility increased from 4% to 6% after treatment with 10 kGy [40]. Under these conditions, its water-holding capacity, oil-holding capacity and cholesterol adsorption capacity also improved. This pathway is limited by factors such as high-cost equipment, strict regulatory requirements, and low acceptance of irradiated ingredients in some markets. In addition, its effective window was narrow because excessive irradiation led to severe degradation and functional decline. In summary, extrusion was most suitable for large-scale food processing, whereas electron beam irradiation had low industrial accessibility with precise control.

### 3.3. Chemical Modification

Chemical modification offers a wider range of means than that of physical modification to regulate AX function. These modifications include etherification, oxidation, and phenolic acid grafting or esterification [41]. These reactions alter hydrophilic–hydrophobic balance, chain mobility, and cross-linking behavior. Cereal bran AXs are inherently heterogeneous in molecular weight, substitution patterns, and the degree of ferulic acid acylation. Accordingly, chemical modification typically separates a series of modified products rather than a single product [42]. Furthermore, chemical treatment introduced issues related to residues, purification, safety, and regulatory compliance, which were particularly important for food applications.

Etherification enhanced the flexibility and processability of the molecule by introducing substituents, such as hydroxypropyl or hydroxyethyl groups, on the Xyl/Ara residues. Lorenz et al. [43] proved that when using cyclic carbonates, particularly propylene carbonate and 4-vinyl-1,3-dioxolane-2-one, for modification, arabinose residues would be preferred for substitution [43]. These results indicated that chemical substitution substantially expanded the application of AXs in packaging and flexible materials. However, the feasibility of direct use in food-grade systems might be limited owing to its reliance on synthetic reagents and subsequent purification.

Through the ring-opening addition reaction of n-butyl ethylene oxide (BuGE), a hydrophobic side chain could be introduced to form a thermoplastic AX ether. Deralia et al. showed that an appropriate amount of BuGE could achieve a one-step reaction to prepare melt-processed materials. These materials were melted and also made into stretchable thermoplastic films via compression molding [19]. These hot-pressed films exhibited approximately 200% elongation at break and typical stress–strain behavior of thermoplastic polymers. To achieve excellent gel and barrier properties, phenolic acid grafting and esterification reactions exploit the inherent cross-linking ability of ferulic acid. For example, N, N’-carbonyldiimidazole activation technology was used to enrich ferulic acid content on arabinose residues. Thus, the critical gel concentration was reduced from 35% to 25% (*w*/*v*) [44]. Moreover, chemical modification provides a flexible strategy. Specifically, chemical modification links the precise structural changes in AXs to specific functions in the fields of food, packaging, and biomedicine.

Even though, reagent toxicity, incomplete removal of activators, high costs, and regulatory uncertainties remain substantial limiting factors. Consumer acceptance may also decrease when non-natural chemical groups are introduced into AXs in large quantities. Overall, chemical modification is a flexible strategy to improve the processability and cross-linking properties of AXs. However, its practical value in food depends on functional enhancements, residue control, safety, cost, and regulatory applicability.

### 3.4. Enzyme Modification

Enzyme modification has unique advantages in the functional regulation of AXs. Compared with chemical modification conditions, enzyme-catalyzed reactions are carried out under mild conditions and have excellent site specificity and stereospecificity. This renders it possible to identify and modify specific sites on the main chain, side chain, and phenolic substituents of AXs accurately. The process also retains the biological activity of polysaccharides. However, its applicability is limited by enzyme cost, reaction time, substrate accessibility, and scalability.

In dough systems, WUAX usually weakens the gluten network by competing for water and inhibiting the polymerization of gluten. Sun et al. found that partial hydrolysis of WUAX using xylanase generated an enzyme-modified fraction known as EAX. This fraction produced a uniform and compact network [45]. Unlike native WUAX, EAX improved loaf volume and texture by strengthening structural integration in high-gluten formulations. Similarly, GH11 xylanase PcXyn11A binds preferentially to heavily substituted cereal AXs. Zhang et al. [46] reported that PcXyn11A broke down wheat AXs with high efficiency, thereby lowering RVA peak viscosity and extending bread freshness. This mechanism substantially improved dough elasticity and water-holding capacity while limiting contact with gluten.

Enzymatic technologies also enhance the mechanical and barrier properties of AX-based films. For example, laccase-catalyzed coupling of ferulic acid to form covalent cross-links improves dimensional stability. Alahmed and Simsek [47] further improved the chemical properties of the film by combining laccase treatment with lipase-catalyzed surface esterification. However, industrial application remains limited by a number of factors. To maintain reproducible enzyme activity, enzymatic processes usually requires strict control of reaction conditions. In complex cereal matrices, the accessibility of AXs and the effectiveness of the enzyme fluctuate with changes in hydration state and solid content, thereby reducing process efficiency [48]. Many natural xylanases suffer from low yield and poor stability, which further limits their wide application in food and large-scale industrial practices.

## 4. Gel Formation and Mechanisms of AXs

**Figure 3:**
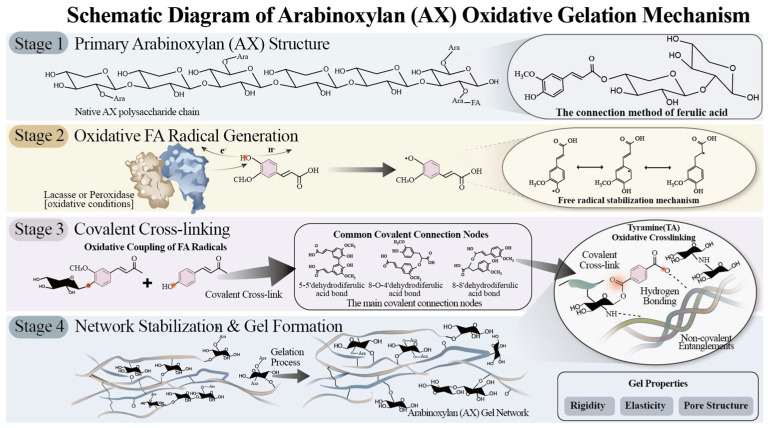
Schematic representation of the oxidative gelation mechanism of arabinoxylan (AX), showing ferulic acid-mediated radical formation, covalent cross-linking, network stabilization, and gel formation.

AX gelation is mainly driven by the oxidative coupling of esterified ferulic acid residues on the arabinose side chain. Under enzymatic or chemical oxidation conditions, phenoxy radicals further form dehydrodiferulic acid cross-links, such as 5-5′, 8-O-4′, and 8-8′ bonds, which constitute the main covalent connection nodes in the network [49]. Thereafter, hydrogen bonds and chain entanglement further stabilize the gel matrix. Figure 3 summarizes this process from ferulic acid radical generation and covalent cross-linking to network stabilization and final gel formation. Gel formation is not controlled by ferulic acid alone. Mechanistically, AX gelation is a continuous process from structure, reaction, to network. Molecular weight and branching characteristics initially determine whether the AX chains fully overlap and entangle, thereby maintaining necessary segmental mobility during network formation [50]. Subsequently, ferulic acid content and oxidase type further determine the degree and rate of free radical-mediated covalent crosslinking. The pH and ions influence further network organization by regulating charge shielding, chain contraction, and non-covalent interactions [51,52]. Ultimately, the rigidity, elasticity, pore structure, and water-holding capacity of the gel essentially rely on the comprehensive balance between chain structure, crosslinking density, and the reaction environment.

### 4.1. Influencing Factors in AXs Gelation

The molecular structure of AXs had a pronounced impact on gel formation. AX gels were not determined by a single parameter, but rather by the presence of segments with sufficient molecular size, moderate branching, and ferulic acid sites in a reaction environment conducive to controlled oxidation and intermolecular association [50]. The A/X ratio and substitution pattern regulated the degree of chain branching, solubility, and intermolecular spacing, whereas molecular weight determined the degree of chain overlap and entanglement [53]. Marquez-Escalante et al. [54] showed that a high degree of substitution aided in maintaining stability between segments and partial debranching reduced the A/X ratio, promoted chain aggregation, and increased the likelihood of ferulic acid crosslinking. Moderate branching was generally the most favorable owing to its ability to achieve a balance between segment density and crosslinking density. Ferulic acid content was also crucial; a high degree of ferulic acylation provided additional oxidative coupling sites, whereas acetyl substitution may shield hydroxyl groups and weaken interchain cohesion [14,55]. Therefore, an effective gel usually results from a balance between sufficient molecular size, appropriate degree of branching, and accessibility to ferulic acid residues.

The oxidation system further determines the gelation kinetics and network structure. Both laccase and peroxidase induced AX gelation; however, the resulting microstructures differed. Yilmaz-Turan et al. [56] found that peroxidase formed a loose and heterogeneous network, whereas laccase formed small aggregates and an elastic gel [56]. The amount of enzyme used was also vital. Insufficient oxidase activity rendered it difficult to establish a continuous network, and excessive oxidation promoted local aggregation and increased brittleness, which resulted in a heterogeneous gel [57]. In addition, pH and some ions regulated the electrostatic environment of AX chains. Specifically, lowering the pH induced chain contraction and enhanced hydrogen bonding after pre-crosslinking, whereas a moderate ionic strength (Ca^2+^) shielded charges and improved rigidity, water retention, and phase stability [55,58,59]. Overall, AX gelation should be considered a structure-dependent process in which molecular structure and reaction environment jointly determine the final gel properties.

### 4.2. Construction of AX Composite and Double-Network Gels

Considering that pure AX gels usually require a high solid content and have limited mechanical strength, composite gels and dual-network gels have received increasing attention. Combining AXs with proteins, polysaccharides, or nanofillers improves the texture, stability, and barrier properties of gels. Protein–AX systems were among the most relevant composite systems for structured foods and delivery matrices [60]. Liu et al. [61] constructed a casein–AX interpenetrating network with enhanced extensibility and shear resistance. Lv et al. [62] prepared a densely interconnected wheat bran AX–soy protein isolate emulsion-filled gel, which was used to encapsulate bio-active substances [62]. Proteins are widely used in food, and such systems have high practicality in structured foods, emulsion delivery, and texture design. However, their application faces challenges such as phase separation, water competition, and the need for precise control of pH and oxidation conditions during processing.

AXs are also reinforced by combination with other polysaccharides or nano-fillers. For example, Lara-Espinoza et al. [63] constructed ferulic acid pectin–AX hydrogels with elastic behavior and dense porous structure. Moreover, starch or cellulose nanocrystals (CNC) further improved network density and mechanical strength. Talantikite et al. [64] showed that CNCs enhanced AX hydrogels through adsorption, hydration bonds, and electrostatic interactions. The practical relevance of these systems is not the same. Starch–AX systems were most suitable for structured foods, whereas CNC-reinforced gels were most likely to be applied in packaging or specialty materials. Filler dispersibility and ingredient acceptance were the most critical issues in food systems. Overall, AX composite gels show promising potential. However, their industrial relevance depends on improvements in network design, the simplicity of formulations, the robustness of processes, and the clarity of their applicability in food, delivery systems, or packaging.

## 5. Arabinoxylans in Food Applications and Selected Emerging Uses

**Figure 4:**
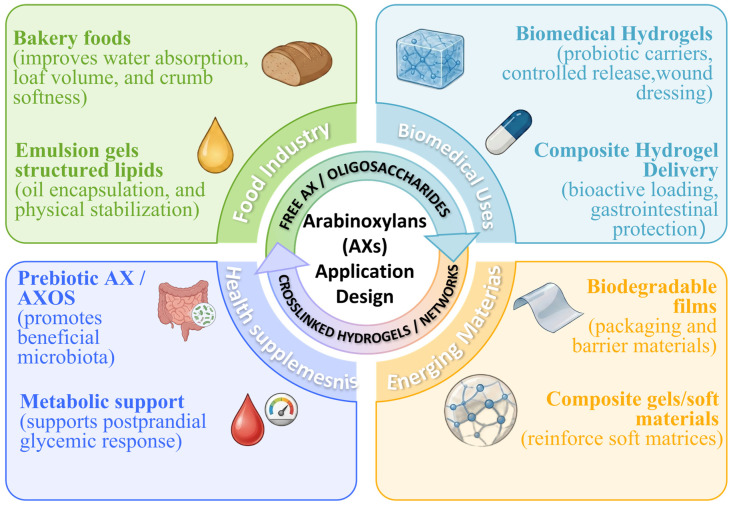
Application domains of AX, highlighting its roles in food systems, health-related functions, biomedical delivery, and emerging material applications.

AXs are employed as a free polysaccharide or oligosaccharide component or a component of structured network. In food systems, free AXs or AXOSs mainly exhibit dietary fiber effects, viscosity regulation, and gut microbiota regulation functions, whereas cross-linked or composite AX networks are most suitable for texture design, encapsulation delivery, and physical stability. These applications are summarized in Table 2, with emphasis on their main functions, forms, limitations, evidence level, and application readiness. Figure 4 provides a comprehensive overview of the application domains of AXs and the primary roles they play.

### 5.1. Arabinoxylans in Food Applications

#### 5.1.1. Dough Rheology and Bakery Applications

Owing to their molecular weight, solubility, and interaction with gluten, different AX fractions have different effects in dough. WEAX at relatively high molecular weights typically have improved water absorption, dough viscosity, gas retention, bread volume, and softness during storage [65,66]. In contrast, WUAX tends to compete with gluten for water and may interfere with gluten network formation. This impairs dough processing performance and product quality unless it is moderately depolymerized. AXOSs generally contribute little to dough structure owing to their limited thickening ability. However, AXOSs still have value as a fermentable dietary fiber component [67]. Enzymatically modified AXs fall somewhere in between. Controlled xylanase treatment mitigates the adverse effects of WUAX and enhances its dispersibility and compatibility with the gluten network to improve bread volume and texture [45,46]. Therefore, the performance in baking systems hinges more on the type of AX fraction than on the AX content itself.

#### 5.1.2. Brewing Applications and Functional Beverages

In the brewing process, AXs are primarily a process-related factor rather than a structural component. Barley-derived AXs elevate wort viscosity, decreases filtration efficiency, promote turbidity, and affect foam properties [68]. Therefore, in practical applications, the molecular weight and AX content are usually controlled rather than utilized for gel formation. In contrast, soluble AXs in beverage systems are most suitable as fiber-rich and prebiotic ingredients [69]. These components increased fiber content and enhanced mouthfeel in fruit juices, dairy beverages, and plant-based beverages while maintaining acceptable viscosity. AXs are also used as a carrier to protect or achieve controlled release of vitamins, flavor compounds, or other sensitive ingredients [51]. However, this usage is substantially less common than employing soluble AXs directly as dietary fiber.

#### 5.1.3. Antioxidant and Interfacial Functions

AXs enhance food system stability through antioxidant activity and interfacial interactions. Esterified ferulic acid residues scavenge free radicals and inhibit lipid peroxidation. Malunga et al. [70] demonstrated that AXs were derived from the wheat aleurone layer and bran inhibited lipid peroxidation in a simulated gastric environment. This effect was closely associated with ferulic acid content and substitution pattern [70]. However, AXs were not a potent small-molecule surfactant. Specifically, their role in dispersion stability primarily depended on interfacial adsorption, continuous phase thickening, steric hindrance stabilization, and interactions with proteins or other macromolecules [71]. In high-water-content foods, cross-linked AX networks further enhance water retention and structural integrity [72]. Thus, the contribution of AXs to oxidative and physical stability is highly system-dependent.

### 5.2. Health-Related Functions and Delivery

AXs and their enzymatic hydrolysis products (AXOSs) are among the most extensively studied cereal-derived prebiotics. In vitro fermentation studies, animal experiments, and review evidence suggest that these components promote the proliferation of beneficial bacteria, especially Bifidobacteria and Lactobacillus, and increased short-chain fatty acid production [73,74,75,76]. Animal experiments also indicated potential anti-inflammatory effects and improved intestinal immune regulation [77]. In practical food applications, free AXs or AXOSs were most suitable for daily supplementation in cereals, powders, or granules. Conversely, when protecting probiotics or achieving local intestinal delivery, AX gel particles or dual-network gels were advantageous.

The relationship between AX and metabolic regulation should be interpreted cautiously. In vitro studies, animal models, and limited human intervention trials suggest that cereal fibers rich in AX benefited postprandial blood glucose response, lipid metabolism, and related metabolic indicators [73,76,78]. Animal studies also showed that AX diets, alone or combined with caffeic acid or β-glucan, reduced oxidative stress, inflammation, and hepatic lipid accumulation [66]. However, these results were insufficient to support broad clinical conclusions. Existing evidence focuses heavily on physiological plausibility and dietary support potential. Moreover, direct clinical evidence remains limited and is influenced by AX source, dosage, food matrix, and study design. Therefore, AXs should be viewed as a dietary component that potentially supports metabolic health rather than as a broadly established therapeutic intervention.

### 5.3. Selected Emerging Non-Food Uses

Although AXs were primarily used in food applications, their biodegradability, low toxicity, and crosslinking potential have prompted exploration in biomedical and materials fields. AX hydrogels have been investigated as probiotic carriers and colon-targeted delivery systems [44,57,79]. Chemically modified or composite AX materials have also shown potential as biodegradable films, coatings, and other sustainable materials [33,80]. Overall, food remains the primary and most practical application area for AXs, whereas biomedical delivery and advanced materials represent promising yet premature expansion directions.

**Table 2:** Representative applications of AX, including main functions, application forms, major limitations, evidence level, and application readiness.

Applications		Main Function	Application Forms	Main Limitations	Evidence Level	Application Readiness	References
Applications in food Industry	Rye/Wheat bread	Improves water absorp-tion, gas retention, loaf volume, crumb softness, and fiber enrichment	Arabinoxylan	Effects depend strongly on AX fraction typ	Food formulation studies	High	[55,81]
	Functional bakery products	Improves extensibility, dough development, and bread quality.	Arabinoxylan, in some systems, forms a local cross-linked network with the help of laccase and other agents.	Benefits depend on controlled depolymerization; over-hydrolysis may reduce structural contribution	Food formulation studies	Medium	[46,66]
	Dough systems	Adjusts rheological properties, extensibility of the dough, and crispnes.	Arabinoxylan	Excess AX may impair spread, texture uniformity, or sensory acceptance	Food formulation studies	Medium	[82,83]
Applications of food and supplements related to health	Emulsion-filled gels and structured lipids	Encapsulates the oil and develop a structured low-fat system.	Arabinoxylan-protein complex hydrogel	Sensitive to phase separation, oxidation and protein-AX compatibilit.	Formulation studies	Medium	[62,84]
	Composite hydrogel delivery system	Protects bioactive substances and regulating the release in the gastrointestinal tract	Arabinoxylan-based composite hydrogel	More relevant to functional delivery systems than conventional foods.	Simulated digestion in vitro and formulation studies	Low-Medium	[63,79]
	Type 2 diabetes intervention	Improves glucose tolerance and lowers post-meal blood sugar levels	Arabinoxylan/cross-linked AXs.	Stronger evidence is still needed	Animal evidence	Low-Medium	[77,85]
	Multiphase composite edible gels	Texture design and edible gel structuring	Arabinoxylan complex edible hydrogel	Requires precise control of pH, oxidation, and component compatibility	Formulation studies	Medium	[64,84]
	Prebiotics and gut microbiota regulation	Improves the formation of beneficial bacterial flora and SCFA.	Arabinoxylan/Arabinoxylan oligosaccharides	Effects depend on molecular size, substitution pattern, dose, and host microbiota	Animals upported evidence	Medium	[73,74,75,76]
	Antioxidant, lipid-lowering, and immune support	Provides antioxidant, lipid-lowering and immune-regulating support.	Arabinoxylan	Many claims rely on model systems, animal studies, or limited human evidence	Limited human evidence	Low-Medium	[70,83,86]
	Comprehensive functional foods	Promote metabolism and intestinal health.	Arabinoxylan	Functional benefit depends on dose, matrix, and processing	Limited health evidence	Medium	[87]
Applications in emerging non-food	Edible and biodegradable films	Packaging and film-related applications.	Cross-linked or plasticized arabinoxylan films	Moisture sensitivity, processing robustness, and cost still need optimization	Formulation studies	Low-Medium	[47,87]
	Biobased soft materials	Constructs enhanced hydrogels for soft materials and advanced manufacturing systems.	Arabinoxylan complex hydrogel	Limited direct food relevance; dispersion, printability, and cost remain constraints	Material concept verification	Low	[64]
	Biomedical hydrogels	Controlled release and tissue-related biomaterials	Arabinoxylan-based biomedical hydrogel	Outside the main food scope; regulatory, cost, and translational barriers remain high	Material concept verification	Low	[56,57,64]

Cite: High: already compatible with current food formulation practice. Medium: promising in model foods, but scale-up or formulation robustness remains unresolved. Low-Medium: supported mainly by in vitro or proof-of-concept studies; practical food use remains limited. Low: mainly exploratory or outside routine food processing practice.

## 6. Challenges and Future Perspectives

### 6.1. Technical and Translational Bottlenecks

Cereal raw materials exhibit considerable variability. AXs from different sources show marked differences in molecular weight, substitution patterns, and ferulic acid content. This variability complicates the comparison of research results and hinders the establishment of stable structure–function relationships. Second, processing-induced structural damage remains a major limitation. As aforementioned, an increase in extraction yield often corresponds to a decrease in molecular weight and inadequate retention of ferulic acid. This weakens the thickening, oxidative cross-linking, gelling, and certain bioactive functions of AXs. A similar situation occurs during modification processes, where enhancements in solubility or processability frequently result in reduced structural integrity. Additionally, formulation design and scale-up production are highly complex, particularly for composite gels and structured systems. Such systems usually require strict control of pH, ionic strength, oxidation levels, and component interactions to achieve stable and reproducible performance. Finally, regulatory and evidentiary deficiencies continue to hinder further commercialization. Currently, AX-related raw materials have approvals limited to specific sources and applications, with many health effects primarily supported by in vitro studies and animal experiments. In summary, raw material heterogeneity, processing-induced structural losses, formulation complexity, and regulatory uncertainties regarding source specificity remain crucial barriers to the industrialization of AXs.

### 6.2. Future Perspectives: Molecular Engineering and Sustainable Valorization

Future research should prioritize establishing clear links between structure, processing, and function. First, precisely associating monosaccharide composition, substitution patterns, molecular weight, ferulic acid distribution, and cross-linking behavior with macroscopic properties, such as viscosity, gel strength, water retention, and delivery performance, is necessary. To achieve this, analytical methods, such as chromatography, nuclear magnetic resonance, mass spectrometry, and in situ spectroscopy should be systematically combined to characterize AX components derived from various extraction and modification methods. Second, scalable and food-grade processing strategies should be developed. These should focus on yield while preserving functional molecules and ferulic acid sites. This is particularly important for applications dependent on gelation, structural control, and interfacial properties. Future research should clearly differentiate between food-grade products, health-supporting components, delivery systems, and non-food materials, as distinct applications require specific structural targets and evidence standards. From a transformational perspective, sustainable and effective utilization of AX-rich byproducts necessitates integrating raw material grading, green extraction methods, targeted modification, application validation, and regulatory positioning. Simply, the future of AXs does not lie in developing increasingly complex modified materials, but rather in identifying structures that can be stably prepared, safely used, and effectively matched with food, health, or material applications.

## 7. Conclusions

AXs are a highly promising class of functional ingredients and structure-forming polysaccharides. However, their practical application depends on effectively preserving key structural features during extraction and modification. Generally, alkaline extraction and SWE are most favorable for achieving high recovery rates, whereas water extraction and partially enzyme-assisted extraction maintain molecular structural integrity and ferulic acid-related functions. For gel formation, oxidative cross-linking of ferulic acid-acylated AXs remains the most effective strategy, particularly under conditions preserving sufficient molecular size and ferulic acid sites. Nevertheless, industrial application of AXs currently faces multiple constraints, including heterogeneity of raw material sources, trade-offs between yield and structural preservation, complexity in large-scale production, and insufficient human evidence supporting many health effects. Accordingly, future research should emphasize preparing AX components with clearly defined structures, reproducible outcomes, strong food applicability, and alignment with specific applications.

## Data Availability

All data extracted from the included studies are presented in the main text. No additional raw data were generated.
